# The Sm14+GLA-SE Recombinant Vaccine Against *Schistosoma mansoni* and *S. haematobium* in Adults and School Children: Phase II Clinical Trials in West Africa

**DOI:** 10.3390/vaccines13030316

**Published:** 2025-03-16

**Authors:** Amadou Tidjani Ly, Doudou Diop, Modou Diop, Anne-Marie Schacht, Abdoulaye Mbengue, Rokhaya Diagne, Marieme Guisse, Jean-Pierre Dompnier, Carolina Messias, Rhea N. Coler, Celso R. Ramos, Jacques-Noël Tendeng, Seynabou Ndiaye, Miryam Marroquin-Quelopana, Juçara de Carvalho Parra, Tatiane dos Santos, Marília Sirianni dos Santos Almeida, Daniella Arêas Mendes-da-Cruz, Steven Reed, Wilson Savino, Gilles Riveau, Miriam Tendler

**Affiliations:** 1 Biomedical Research Center Espoir Pour La Santé, Saint Louis BP 226, Senegal; 2 CIIL-Center for Infection and Immunity of Lille, Institut Pasteur de Lille, University of Lille, CNRS UMR, Inserm U1019-Lille, F-59000 Lille, France; 3 Laboratory on Thymus Research, Oswaldo Cruz Institute, Oswaldo Cruz Foundation, Rio de Janeiro 21040-360, Brazil; 4 Brazilian National Institute of Science and Technology on Neuroimmunomodulation, Oswaldo Cruz Institute, Oswaldo Cruz Foundation, Rio de Janeiro 21040-360, Brazil; 5 Seattle Children’s Research Institute, Center for Global Infectious Disease Research, Seattle, WA 98105, USA; 6 Department of Pediatrics, University of Washington School of Medicine, Seattle, WA 98105, USA; 7 Department of Global Health, University of Washington, Seattle, WA 98105, USA; 8 Fenix Biotec Treinamento SS LTDA, São Paulo 05591-090, Brazil; 9 Hopital Régional de Saint Louis, UFR 2S, Université Gaston Berger, Saint Louis BP 226, Senegal; 10 Région Médicale de Saint Louis, Ministère de la Santé et de l’Action Sociale, Saint Louis BP 226, Senegal; 11 Laboratory of Anti-Helminth Vaccine Research and Development, Oswaldo Cruz Institute, Oswaldo Cruz Foundation, Rio de Janeiro 21045-900, Brazil; 12 Rio de Janeiro Research Network on Neuroinflammation, Oswaldo Cruz Institute, Oswaldo Cruz Foundation, Rio de Janeiro 21040-360, Brazil; 13 INOVA-IOC Network on Neuroimmunomodulation, Oswaldo Cruz Institute, Oswaldo Cruz Foundation, Rio de Janeiro 21040-360, Brazil; 14 HDT BioCorp, Seattle, WA 98102, USA; 15 FABP Biotech, Rio de Janeiro 22611-100, Brazil

**Keywords:** vaccine, schistosomiasis, Sm14 protein, phase II clinical trials, safety, immunogenicity, adults, school-age children

## Abstract

Background/Objectives: Following previous successful Phase I clinical trials conducted in men and women in a non-endemic area for schistosomiasis in Brazil, the Sm14 vaccine was evaluated in an endemic region in Senegal. We report successful clinical trials in adults (Phase IIa) and school children (Phase IIb), respectively, of a *Schistosoma mansoni* 14 kDa fatty acid-binding protein (Sm14) vaccine + a glucopyranosyl lipid A (GLA-SE) adjuvant. Methods: Participants were evaluated based on clinical assessments, laboratory tests (including hematologic and biochemical analyses of renal and hepatic functions), and immunological parameters (humoral and cellular responses) up to 12 months after the first vaccination dose in the Phase IIa trial and after 120 days in the Phase IIb trial. Results: The results showed strong immunogenic responses and good tolerance in both adults and children, with no major adverse effects. Importantly, significant increases in Sm14-specific total IgG (IgG1 and IgG3) were observed as early as 30 days after the first vaccination, with high titres remaining at least 120 days afterwards. Sm14-specific total IgG serum levels were also significantly enhanced in adults and in both infected and non-infected, vaccinated children and elicited robust cytokine responses with increased TNFα, IFN-γ, and IL-2 profiles. Conclusions: Overall, the Sm14+GLA-SE vaccine is safe and highly immunogenic, with a clearly protective potential against schistosomiasis, supporting progression to the next Phase III clinical trials.

## 1. Introduction

Schistosomiasis is a parasitic disease caused by worms of the genus *Schistosoma*, which is present in tropical areas, particularly in sub-Saharan Africa, parts of Latin America, and Southeast Asia. The World Health Organization (WHO) regards it as a neglected tropical disease (NTD) and estimates that over 240 million people in more than 70 countries are infected, with around 800 million living in endemic areas, reflecting the need for global programs directed at the effective control of schistosomiasis transmission [1]. Considering the size of the global territory affected and the number of people affected, there is no doubt that schistosomiasis has a hugely negative public health impact. Many countries are endemic for only one of the main three (and the few other species of this parasite), such as *S. japonicum* in China, The Philippines, and Indonesia, with *S. mansoni* in sub-Saharan Africa and in different geographical regions in Brazil. The African countries not only harbour about 90% of the burden due to this infection, but most of them are endemic for both *S. mansoni* and *S. haematobium*. The lower Senegal River Basin is one of the world’s most highly endemic areas and is characterized by high transmission rates [2]. It should be added that *S. mansoni* and *S. haematobium* cause intestinal schistosomiasis, while *S. haematobium* infections result in urogenital disease.

With the exception of *S. japonicum*, which can infect a variety of mammals, humans are the definitive host for this parasite, whose life cycle includes different freshwater intermediate snail hosts. Infected snails produce and release the infective stage (cercariae) that threatens humans during water contact in the endemic areas. The snail was once the focus for countering transmission of the disease, but after the development and positive clinical trials of praziquantel in the 1970s [3], the approach switched to mass drug administration (MDA) of this drug using treatment cycles with intermissions of various lengths, generally a year. Clearly, this curative anti-morbidity strategy needs a long-term counterpart to avoid endless repeated treatments, and efforts to achieve this with the inclusion of a vaccine has been ongoing for more than 30 years [1,4]. Recognizing that the interruption of schistosomiasis transmission through preventive drug therapy alone is ineffective, various combined strategies (in which snail control is making a recent comeback) are now contemplated, where vaccination is increasingly seen as key [5].

Out of the few existing vaccine candidates for a schistosomiasis vaccine, the Sm14 molecule remains strong, as shown in our previous work demonstrating that immunization with a soluble extract from *S. mansoni* protects mice and rabbits against the infection [6,7]. The active molecular component in this extract was identified as a 14 kDa member of fatty acid-binding proteins with a defined three-dimensional structure [8]. We identified the gene encoding this protein and were subsequently able to express large amounts of recombinant Sm14 (rSm14) protein using various biological platforms [9,10,11]. Regarding recombinant vaccines, there are major advantages. The main advantages, particularly for a protein antigen such as Sm14, are that the recombinant process enables the production of highly purified vaccines, making them much safer and significantly more cost-effective. It is a reproducible method that, among all the currently available methods for next-generation vaccines, makes recombinant vaccines the best choice. They are the most widely used, the safest, the most cost-effective, and are highly standardized, especially in the context of protein-based vaccines. Additionally, the fact that recombinant vaccines are widely used means that there is extensive experience with them, which is a significant advantage in terms of safety.

Phase I clinical studies in adults from a non-endemic area of Rio de Janeiro, Brazil eventually showed Sm14 plus the adjuvant glucopyranosyl lipid A in squalene emulsion (GLA-SE) to be safe and strongly immunogenic [12,13]. We aimed to advance the research on this vaccine candidate by studying its safety and immunogenicity in adults and school children living in an endemic area in Senegal characterized by high transmission, and we report here the successful results of Phase II clinical trials in adults and school children [14,15].

## 2. Materials and Methods

### 2.1. Vaccine Antigen and Adjuvant

The vaccine composition and antigen used in both Phase IIa and IIb was the same cGMP lot (PBR-0057-002) produced at Cornell University, Ithaca, NY, USA, used in the Phase I trials. The glucopyranosyl lipid A adjuvant in squalene emulsion (GLA-SE) was supplied by the Infectious Diseases Research Institute/Access to Advanced Health Institute (AAHI/IDRI), also the same lot (Part # 0037-09M001) as in the Phase I trials [12,13].

### 2.2. Location and Study Cohorts

The study was carried out in the lower Senegal River Basin, northern Senegal (Figure S1), which is endemic for both *S. mansoni* and *S. haematobium*. Here, density and prevalence of the specific intermediate snail hosts are high, and reinfection in school children, particularly by urogenital schistosomiasis, is common soon after preventive chemotherapy [3]. The study was registered within the local platform ClinicalTrials.gov (https://clinicaltrials.gov/); one for adult participants (Phase IIa) [14] and one for school children (Phase IIb) [15]. Children of both genders, aged from 8 to 11 years old, were recruited from three villages: Sor Diagne, Pont-Gendarme, and Ndiagambal, while all adult subjects came from Pont-Gendarme. A particularity regarding their infections was the quasi-specificity of the *Schistosoma* strain in each location; children in Pont-Gendarme (n = 32) were mainly infected by *S. mansoni*, while those in Ndiagambal (n = 48) were mainly infected by *S. haematobium*. Although most had a single-species infection, a few children (n = 10) were infected by both *S. mansoni* and *S. haematobium*. Children from Sor Diagne were uninfected and in generally good health.

For the Phase IIa trial, two cohorts of 15 male volunteers were selected from residents who had previously had schistosomiasis and been treated and who were currently free from infection. For the Phase IIb trial, the study comprised three groups: (a) healthy children (n = 15); (b) infected children (n = 40); and (c) infected children who were not vaccinated, but were kept as controls (n = 40). It included 42 male and 53 female children in all.

### 2.3. Clinical and Laboratory Safety Assessment

Vaccine safety examination included observation of inflammatory responses such as pain and swelling at the injection site, regional symptoms (heaviness or pain with passive or active movement of the injected limb), and systemic symptoms (fever > 38 °C, headache, drowsiness, irritability, loss of appetite, chills, myalgia, and arthralgia). Assessments were made by clinical trial physicians 1 and 4 h after administration of each dose of the vaccine as well as 24 and 48 h later. The full safety assessment included medical consultations with complete physical examinations and laboratory tests before and after the start of the trial on Day 0 and at Weeks 1, 4, 5, 8, 9, 12, and 16. Participants were assessed in person at a medical consultation 1 h after injection and were contacted by telephone approximately 24 h later. All adverse events (AEs) were classified by severity as Grade 1 (easily tolerated, minimal discomfort not interfering with daily activities); Grade 2 (discomfort preventing normal activities but requiring no or minimal treatment); Grade 3 (preventing normal activities and requiring treatment); and Grade 4 (life-threatening).

The safety outcomes in the Phase IIa and Phase IIb trials of the Sm14 vaccine were defined and assessed following rigorous international regulatory and ethical standards to ensure the reliability and validity of the results. The justification for the chosen criteria is based on the following key factors:

Compliance with International Guidelines

The trials strictly adhered to the International Centers for Tropical Disease Research Network (ICTDR) manual [16] in the United States, ensuring alignment with globally recognized safety assessment protocols. Additionally, all procedures complied with US Federal Drug Administration (FDA) guidelines [17], which establish gold-standard methodologies for evaluating vaccine safety and tolerability.

Accredited Laboratory Testing

The local laboratory responsible for the safety assessments is certified by the International Proficiency Program for all relevant tests, as per the College of American Pathologists (CAP). This certification guarantees that all laboratory analyses met the highest international standards for accuracy and reliability.

Independent Safety Oversight

To ensure unbiased safety monitoring, a Data Safety Monitoring Board (DSMB) was established, composed of health authorities from Senegal. The DSMB was responsible for reviewing, approving, and overseeing the progression of the trials, providing an independent verification of safety outcomes at each stage.

Certain AEs, i.e., those due to accidents or common infections, were logged but not counted as side effects attributable to the product or the operation of the clinical trials.

The proportion of AEs and 95% confidence intervals (CIs) were calculated using SPSS software, version 24 for Mac based on the Clopper–Pearson method [18]. Given that the probability of the occurrence of an AE is always greater than zero, CIs were calculated even when no event had been observed. The CI can then be interpreted as a measure of precision of the estimated probability that an event actually occurs. For example, even if no serious adverse event occurred after the first dose (estimated probability = 0), a serious AE could still occur in a larger population (probability > 0), but that probability must be deemed exceedingly small. Since no group included more than 15 participants, the CI values presented correspond to the observation of 0 events among 15 participants and could therefore vary from 0 to 0.218 (corresponding to the range 0–21.8% of the people who received the vaccine). This means that if a large number of studies with 15 people were conducted under the same circumstances, one would expect that 95% of the studies would produce CIs covering the true value of the probability of a serious AE occurring in the whole population.

During the run-in phase baseline, as well as during the trial, regular laboratory tests were carried out one week after each vaccine injection (Weeks 1, 5, and 9) to ensure the health of the study subjects. Complete blood counts were carried out, with liver and kidney functions monitored during the post-immunization follow-up phase in Weeks 13, 7, and 21. For each of these laboratory tests, a 10 mL sample (from adults) of venous blood was taken: 5 mL in a dry tube and 5 mL in an EDTA tube. For children, following the Senegalese Ministry of Health (MoH) guidelines, a 4 mL sample was taken at each time point (Table S1 shows the types of tests included). The test analyses were performed in a certified clinical laboratory in St. Louis, Senegal.

Statistical analyses were performed for systemic and local reactogenicity at 1 h, 2 h, 24 h, and 48 h after each of the three vaccinations given. Results of the biochemistry and haematologic evaluations were compared with the reference values given by the Laboratoire Regional de Biologie Medicale (LRBM) of Saint-Louis and the International Centers for Tropical Disease and Research (ICTDR) as well as the WHO toxicity grading scale.

### 2.4. Investigation of Immune Responses

For the adults, adaptive immune responses induced by the rSm14 vaccine candidate after each injection were measured on Day 0 as well as 4 and 8 weeks later and then during the post-immunization follow-up after 12, 16, and 20 weeks. For the determination of peripheral blood mononuclear cells (PBMCs), 20 mL of venous blood was collected in tubes containing heparin during the baseline studies and on Days 30, 60, and 90. The PBMCs were separated by gradient centrifugation at room temperature on Ficoll-Paque Plus (GE Healthcare, Marlborough, MA, USA), counted, and then cryopreserved until use in liquid nitrogen at −196 °C (reached by stepwise cooling) with foetal calf serum (FCS) (Life Technologies, Carlsbad, CA, USA) and 10% DMSO (Thermo Fisher Scientific, Waltham, MA, USA) at a concentration of 5 × 10^6^/mL.

For the children, samples were collected at three distinct times: Days 0, 84, and 148. After density gradient centrifugation on PanColl human, 1.077 g/mL (PAN-Biotech, Aidenbach, Germany), PBMCs were counted, frozen, and stored in a medium containing 90% of FCS from Dominique Dutscher SAS (Bernolsheim, Germany, https://www.dutscher.com/) and 10% DMSO. Cell vials were kept for 24 h in DutscherCoolCellFreezingBoxes (ref: 830015) at −80 °C and then transferred to liquid nitrogen at −196 °C for long-term storage. Thawing was performed in a 37 °C water bath with the cell suspension added to the RPMI-1640 medium (LGC Biotechnology, Teddington, London, UK), containing 20% of foetal bovine serum (FBS) from Gibco, Thermo Fisher Scientific, and then centrifuged at 400× *g* for 5 min. The supernatants were discarded, and the pellets were re-suspended in 1 mL of thawing medium. Cell counting was performed by microscopy using trypan blue in a CellDrop BF automated cell counter (Denovix, Wilmington, DE, USA).

Measurements for specific anti-Sm14 antibodies were carried out regularly using direct ELISA, marking the day (D) and visit numbers (V) as follows. In the Phase IIa trial with previously infected adults, they were performed at D-0/V-1 (background), D-28/V-2 (after the first immunization), D-56/V-3 (after the second immunization), D-84/V-4 (one month after the third immunization), and then followed up 5 months (D-110/V-5) and 6 months (D-140/V-6) post vaccination. An extension of the trial (Sm14-IIa extension) was carried out to further track the antibody response at 9 months (D-260/V-7) and 12 months (D-360/V-8) post vaccination.

In the Phase IIb trial, the antibody measurements and visiting times were performed at D-0/V-1 (background), D-84/V-4 (1 month after the third immunization), and then followed up 6 months (D1-48/V-6) and 12–14 months (D-450/V-7) post vaccination.

The specific vaccine antigen (rSm14) was coated into the 96 wells of Nunc immuno plates (F96 Cert., Maxisorp, Roskilde, Denmark) at a concentration of 4 µg/mL in carbonate–bicarbonate buffer (pH 9.6) for 1 h at 37 °C. Afterwards, the plates were filled with phosphate-buffered saline containing 0.5% gelatine (Merck, Darmstadt, Germany) for 1 h at 37 °C, after which serial dilutions (with TpD: phosphate-buffered saline with 0.05% Tween and 0.1% gelatine) of serum samples were added and the plates incubated for 1 h at 37 °C. Monoclonal antibodies specific to human IgG, labelled with horseradish peroxidase (HRP) (Sigma, Burlington, MS, USA, ref: A8419), were added at 1/60,000 dilution and left for 1 h at 37 °C. Colorimetric development was performed with tetramethylbenzidine (TMB) substrate (Sigma, ref: T0440) using 100 µL/well for 15 min and the reaction was stopped with 50 µL/well of stop solution (Sigma ref: S5814-100 mL). The optical density (OD) was measured at 450 nm in a BioTek EL 808 reader from Agilent Technologies (formerly BioTek Instruments, Winooski, VT, USA). At each step of the ELISA procedure, the plate washing was performed using the plate washer (BioTek Winooski, VT, USA, ELx50).

Only IgG antibody responses were tested in the adult serum samples (Phase IIa), while those in the children (Phase IIb) were also analysed for isotypes IgG1, IG2, and IgG4 using the antisera LS-C351371 diluted 1/6000, Invitrogen-MH1732 diluted 1/1000, and Invitrogen-MH1742 diluted 1/1000, respectively.

The specificity threshold depending on the isotype studied with the vaccine protein was defined as the OD value obtained with the first negative dilution of a reference pool of negative sera. Accordingly, the threshold for IgG was 150, for IgG1 100, and for IgG3 and IgG4 30. These values correspond to the minimum threshold below which results were considered equal to zero. Any vaccinated subject having a titre above the threshold was considered a positive responder to the Sm14 antigen.

### 2.5. Investigation of Cell-Mediated Immunity

We performed an evaluation of PBMC subpopulations, including CD4^+^ T cells, CD8^+^ T cells, B cells, and monocytes. The frequency of functional T cells producing cytokines in response to the rSm14 antigen was assessed by (a) the Luminex method [19,20] and (b) intracellular flow cytometry [12].

In the flow cytometry approach, five panels were established to evaluate the different PBMC subpopulations, their activation and memory T-cell profiles, as well as the cytokines (IFN-γ and TNF-α) produced (Table S2). The evaluated subpopulations and the markers that defined each subpopulation are described in Table S3. For antibodies, isotype control, and dye specifications see Table S4.

PBMC samples were transferred to U-bottom 96-well plates, with the cells stained directly after they had been thawed and washed with the staining buffer from BD Bioscience (Franklin Lakes, NJ, USA). The cells were incubated for 10 min at room temperature (while protected from light) with 50 µL of BD Bioscience’s mixture of human Fc Block™ reagent (designed to reduce potential non-specific antibody-generated staining caused by IgG receptors in various applications) and fixable viability stain (FVS) 520. The cells were then washed twice with the staining buffer, incubated with 10 µL of a mixture of five different antibodies (one for each panel) for 30 min at 4 °C and protected from light, washed twice, and then incubated for 20 min with 2% formaldehyde. After centrifuging the plates for 5 min at 400× *g*, the pellets were finally re-suspended in the staining buffer. Samples were kept at 4 °C until being investigated in an FACS Celesta flow cytometer (BD Bioscience). The FVS520 solution and antibodies were diluted to optimal concentration in the staining buffer.

The intracellular cytokine staining was performed using the Cytofix/Cytoperm Plus™ fixation and permeabilization kit (Franklin Lakes, NJ, USA) with BD GolgiStop™ protein transport inhibitor containing monensin, which accumulates the cytokines produced inside the cell. After centrifugation for 5 min at 400× *g* in U-bottom 96-well plates, the cell pellets were re-suspended in the activation medium containing 10 µg/mL rSm14 + monensin + RPMI-1640 containing 10% FBS, with 40 ng/mL PMA + 4 µg/mL ionomycin + monensin + RPMI-1640 containing 10% FBS as positive control. The plates were incubated for 4 h at 37 °C in an atmosphere containing 5% CO_2_ and then centrifuged as before. The cells were then washed and incubated with the Fc-Block/FVS520 solution as described above. The cells were then washed twice with stain buffer and incubated for 30 min at 4 °C (while protected from light) with a 10 µL extracellular antibody mixture directed against CD3 and CD8 receptors. Subsequently, the cells were washed twice with the staining buffer and incubated with the fixation and permeabilization solution for 20 min at 4 °C under light protection. The cells were then washed twice with 1X BD Perm\Wash™ buffer, after which 10 µL of an intracellular antibody mixture directed against CD4, IFN-γ, and TNF-α (alternatively, against CD4 and isotype controls) was added to the different wells and incubated for 30 min at 4 °C while protected from light. The cells were washed twice with 1X BD Perm\Wash™ buffer and incubated for 20 min with 2% formaldehyde, after which the plates were centrifuged for 5 min at 400× *g* and the pellets re-suspended in the staining buffer. The Fc Block/FVS520 mixture and the extracellular antibody mixture were diluted in the staining buffer and the intracellular antibodies mixture in the 1X BD Perm\Wash™ buffer. The cells were kept at 4 °C (no longer than 48 h) before they were investigated in an FACS Celesta flow cytometer (BD Bioscience), with the data analysis performed through FlowJo Software (BD Bioscience, https://www.bdbiosciences.com/en-us/products/software/flowjo-v10-software, accessed on 14 January 2025). The adopted flow cytometry-based gate strategy is shown in Figure S2.

The statistical analysis for these experiments was performed using Graph Pad Prism 7 software (https://www.graphpad.com) through the paired and non-parametric Friedman test [21] followed by the Dunn’s post-test [22] or through the paired and non-parametric Wilcoxon test [23]. Statistical significance was measured at four levels: *p* < 0.05 (*), *p* < 0.01 (**), *p* < 0.001 (**), and *p* < 0.0001 (***).

### 2.6. Vaccination of Adults—Phase IIa

**Figure 1:**
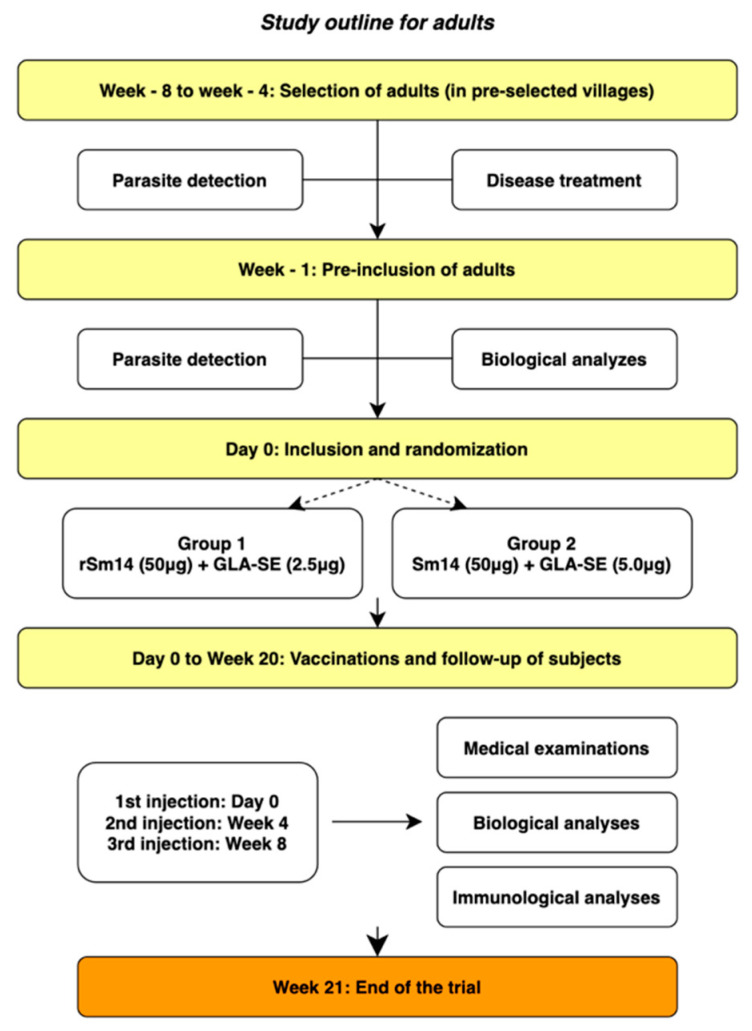
Protocol for vaccination of Senegalese adults (Phase IIa trial).

Two cohorts of 15 male volunteers, 18–49 years of age in each, were given 50 μg rSm14 vaccine candidate, one together with 2.5 µg of the GLA-SE adjuvant and the other with 5 μg of the adjuvant. The trial was open-label, randomized, and carried out including participants with an infectious history of intestinal and/or urinary schistosomiasis. The subjects were treated before entry with 40 mg/kg of praziquantel at least 3 weeks prior to the first injection of the vaccine. After the first injection on Day 0, boosters were given 4 weeks and 8 weeks later (Figure 1). Praziquantel (40 mg/kg single dose) was administered to all participants in the study before vaccination to ensure absence of infection. Of note, upon entry in the clinical trial, the volunteers did not present with any ongoing schistosomiasis infection according to the Kato–Katz test (stool) and the urinary filtration method (urine) [24].

Each injection was preceded by safety measurements applied progressively, with follow-up 1 h and 4 h after each injection, including medical visits at 24 h and 48 h post injection. Three further monitoring visits were carried out after the third vaccination, i.e., on Weeks 12, 16, and 20. This part of the study did not include a control cohort, and the results reported were provided only for those subjects receiving 50 μg of rSm14 together with 2.5 μg of GLA-SE. Finding that responses were similar or even stronger with 2.5 µg of the adjuvant (Table S5) in the first part of the study, the lower dose of the adjuvant was selected for the school children as well as for further studies performed in the adults. This move was supported by previous data from leishmaniasis and tuberculosis trials, indicating that immune responses induced by both doses of the adjuvant were not significantly different [25,26,27,28].

#### 2.6.1. Vaccination of Adults—Extended Phase IIa

After completion of the Phase IIa trial with serological evaluation 6 months after the first rSm14 vaccine dose, and considering the high antibody titres observed, the Senegalese MoH approved [14] an extended evaluation of the antibody response by assessing the persistence of the specific immune responses 9 and 12 months after the first vaccine dose.

#### 2.6.2. Vaccination of Children—Phase IIb

**Figure 2:**
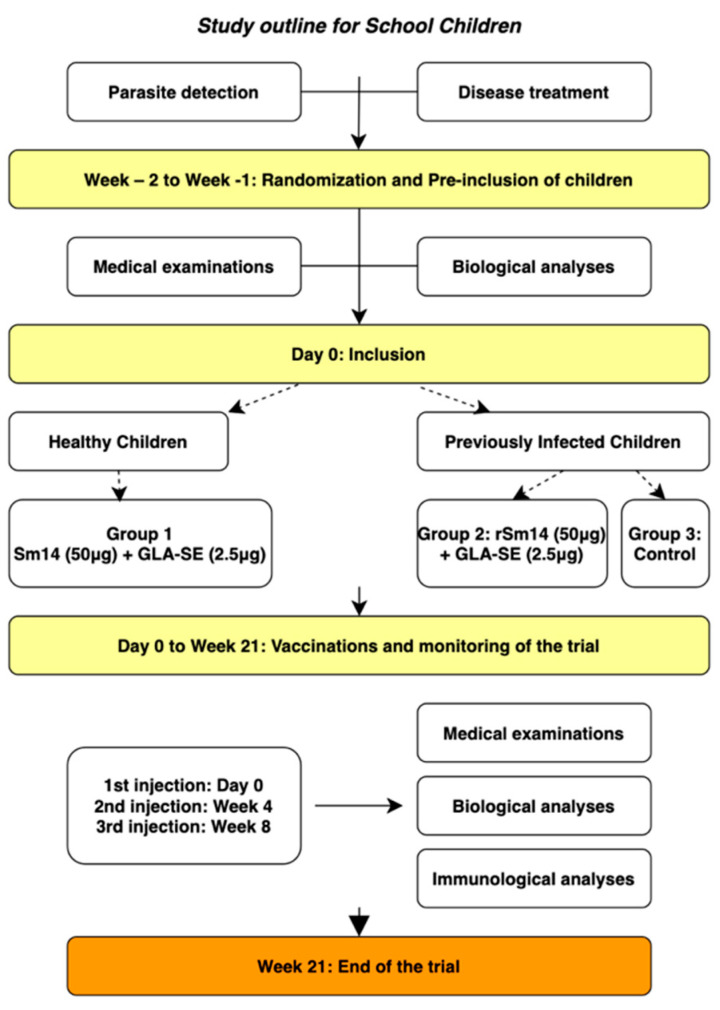
Protocol for vaccination of Senegalese school children (Phase IIb trial).

Table 1 shows the characteristics of the children in the three groups. Each individual in the first two groups received three injections of 50 µg rSm14 with 2.5 µg GLA-SE, with those in the third group left unvaccinated as controls. The outline of the study (Figure 2) shows the protocol, which included treatment with praziquantel 8–14 days before first vaccination followed by two further injections at 1-month intervals. Evaluation of each parameter was carried out on Day 0, and at 1, 3, and 12 months after the third injection.

#### 2.6.3. Safety-Related Measures

A similar strategy as that adopted for the adults was adopted. In the interest of safety, inclusion of children and assignment of treatment arms were performed in an increasing and progressive manner. As shown in Table 2, we began vaccinating healthy children, who all received their first vaccine injection on the same day, and ended with those who were infected. The first vaccine injections in the latter group were administered when all healthy children had received their first vaccination. This part was carried out over a total of approximately 7 weeks.

## 3. Results

### 3.1. Adult Trial (Phase IIa)

#### 3.1.1. Safety Outcome

No serious AEs were observed in the adults. Table 3 shows the recorded proportions with 95% CIs for the various AEs recorded. The most common reaction was local pain in the area of the vaccine injection (9 out 10 participants reported this after the first dose), with decreasing proportions after each subsequent vaccination (5 out 9 for the second dose and 4 out of 10 for the third). In a single instance, a few minutes of minor, involuntary muscle contractions were observed after the first dose of the vaccine. Two participants had fever on Day 7, with two others experiencing headache and light local hyperaemia in the vaccinated arm on Day 37, effects whose association with the vaccination, although improbable, cannot be ruled out. Physical examinations during the baseline and on Days 0, 7, 30, 37, 60, 67, 90, and 120 after the first injection showed no clinical alterations or abnormalities. Laboratory examinations did not show changes that could be classified as toxicity according to ICTDR criteria, except for alanine aminotransferase (ALT) and aspartate aminotransferase (AST), which were slightly elevated in a few participants but without reaching pathological levels. As these participants also had similar elevated values prior to the vaccination (at baseline), and since they had no other abnormal laboratory findings or clinical symptoms, this observation was not considered to be related to the vaccine.

#### 3.1.2. Humoral Immunity

**Figure 3:**
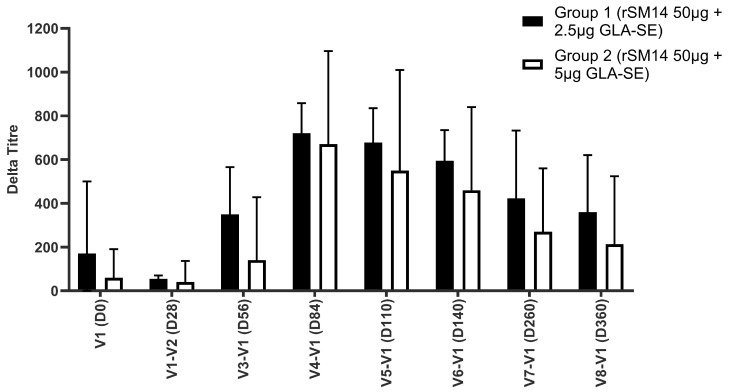
IgG anti-Sm14 responses after vaccination of Senegalese adults. Group 1 (n = 15) received three administrations of 50 µg of recombinant Sm14 + 2.5 µg GLA SE with antigen with one-month intervals. Group 2 (n = 15) received three administrations of 50 µg of recombinant Sm14 + 5 µg GLA SE with one-month intervals (*p* < 0.05).

Regardless of the amount of adjuvant (2.5 or 5.0 µg GLA-SE) used, there was an increase in total IgG over time after vaccination, although the increase was kinetically faster in the group receiving the lower-dose amount of adjuvant, as seen in Figure 3. Although the responses on Day 28 were relatively uniform, the increases measured on the remaining days were all higher for the vaccine doses containing the lower adjuvant amount. Values peaked around Day 84 and decreased slightly after that.

The results of the total IgG production are presented as delta titres, which (= the titre at time X − the titre observed at D-0/V-1, and which presents the effect of vaccination most clearly). The maximum response was obtained at V-4, one month after the third administration of the vaccine. In terms of isotype, the highest titres were obtained for IgG1 and IgG3. After V-4, the response decreased but persisted nevertheless one year after the first administration of the vaccine. From V-3 and until the last measurement at 1 year, Group 1, receiving 2.5 µg of GLA SE adjuvant, presented a humoral response statistically superior to Group 2, receiving 5 µg of GLA SE (Figure 3). The statistical analysis software (SAS), version 9.1 (SAS Institute, Cary, NC, USA) was used, with statistical significance defined as a two-tailed *p* < 0.05. This “sub-study” allowed us to definitively define the dose of adjuvant (2.5 µg) to be used with the rSm14 antigen (50 µg per dose). This formulation was used in all the following clinical trials.

#### 3.1.3. Cell-Mediated Immunity

**Figure 4:**
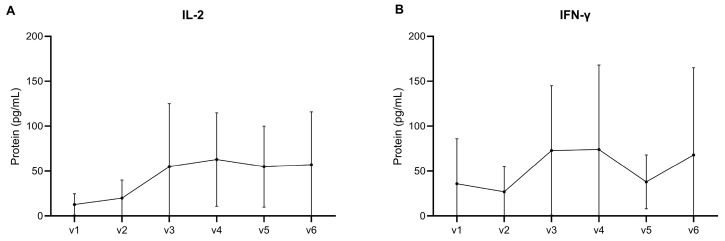
Cytokine levels IL-2 (**A**) and interferon gamma (**B**) after stimulation in PBMC cultures of samples from 30 vaccinated subjects. The number of days post vaccination (D) were the following: V-1 = D-0; V-2 = D-28; V-3 = D-56; V-4 = D-84; V-5 = D-110 and V-6 = D-140).

Similarly to the humoral immune response, in both groups with different amounts of adjuvant, the Sm14-specific cytokine response was characterized by a predominant increase in IL-2 and IFN-γ after vaccination, as seen in Figure 4A,B. This increase was particularly strong at Day 56 (V3) of the study, when the third dose was applied, and the level stayed elevated until the end of the observations on Day 140 (V6) except for a slight decrease in IFN-γ.

### 3.2. Phase IIb

#### 3.2.1. Safety Outcome

Vaccination in school children with 50 µg rSm14 with 0.25 µg of GLA-SE adjuvant was regarded as safe even if the children reacted slightly more strongly than the adults after the injections. No serious AEs were observed. Table 4 shows the proportions of all AEs recorded. The most common reaction was local pain in the area of the vaccine injection (2 out 40 of the infected children reacted after the first dose and 3 after the second and third doses), with a low proportion of the participants reporting swelling and heavy feeling in the arm, but only after the third injection. Fever, headache, abdominal pain, and vomiting also showed low proportions and were mainly seen after the first vaccination. All AEs noted were of Grade 1 (very mild), except vomiting (Grade 2), which occurred in two participants: one after the first injection and one after the second. All other reactions outside of the normal range were evaluated, and since no abnormal laboratory findings or clinical symptoms were recorded, vaccination of the children was deemed to be without systemic effects.

#### 3.2.2. Humoral Immunity

The IgG immune response was measured at four points in time: (1) baseline, i.e., the time from enrolment up to the start of vaccination; (2) Day 84 (Week 12, or approximately 3 months); (3) Day 148 (Week 21, or approximately 6 months); and (4) Day 400+ (approximately 12 months after the baseline).

IgG should increase if the vaccine is producing the expected immunological response. Each child was classified as a “responder” at a specific time if his/her IgG was greater than 150.

Comparison of the results from the three groups shown in Table 5 indicate that the distribution of responders at the baseline was not associated with the group (*p* = 0.40). After vaccination, however, they were each time. Starting on Day 84 and up to Day 400+, at least 93% of the healthy, vaccinated children and 74% among the infected, vaccinated ones were classified as responders. Among the infected non-vaccinated ones, no more than 33% were responders. The detailed observations for each group follow below:

**Healthy, vaccinated children**: Almost all children had values of IgG below 150 before the first dose of the vaccine. At Day 84, about one month after receiving the third dose, the minimum observed IgG was 1763, and at Day 148, it was 920. At the 12-month follow-up (Day 400+), only 1 child had IgG less than 150. In general, the individuals in this group had a large increase in IgG after the three doses and a gradual decrease in IgG over the subsequent follow-up time. However, at the end of the follow-up, only one child had an IgG value below 150.

**Previously infected, vaccinated children**: Most of these children already had IgG values greater than 150 at the baseline (probably due to previous infections). In general, all individuals showed increased IgG levels after the three doses of the vaccine. However, 3 children were not “responders” at day 84 (or later). Most children had decreased IgG at Day 148, but only 4 of them were not responders at that time. However, at Day 400+, 26% of the children (n = 12) had IgG levels below 150.

**Previously infected, non-vaccinated children**: At the baseline, 20% of the non-vaccinated children had IgG levels greater than 150 (probably due to previous infections). As expected, the IgG levels in this group changed little throughout the study period. However, 5 children with lower IgG levels than 150 up to Day 148, had values greater than that on Day 400+ (however, it cannot be said with certitude that those were due to new infections).

**Figure 5:**
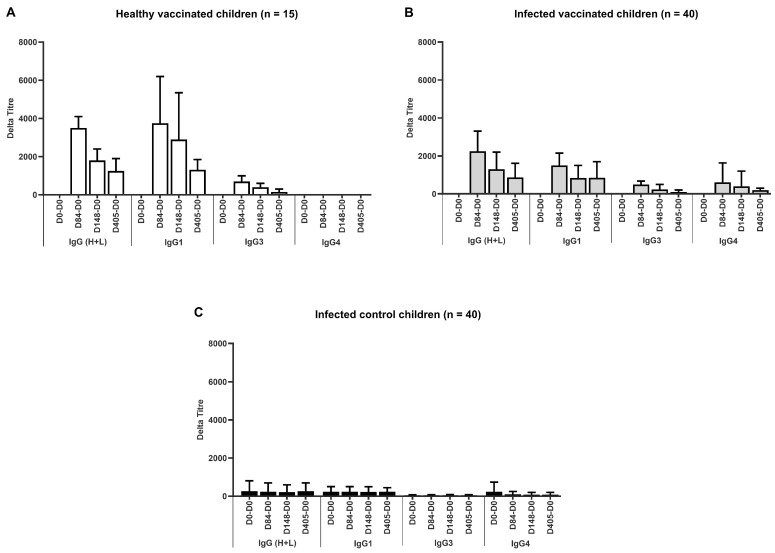
**Anti-Sm14 immunoglobulin responses after vaccination of Senegalese school children.** (**A**) Healthy school children vaccinated and living in St. Louis City, where schistosomiasis is absent (n = 15); (**B**) vaccinated infected children (n = 40); (**C**) non-vaccinated infected children (control group) (n ≥ 40). All infected children were living in areas endemic for *S. mansoni* and/or *S. haematobium*.

Figure 5 shows the specific immune response against rSm14 during one year after the first vaccine injection. For the vaccinated healthy children (Group 1) and vaccinated infected children (Group 2), the results are presented as delta titres (titre at time Tx–titre at T0).

For non-vaccinated infected children (Group 3), the results are expressed as titre means because the titre at D-0 was higher than at later observations, which would make all values of delta titres negative for IgG and the isotypes.

The majority (94%) of children who received the vaccine showed a specific immune response, distributed between Group 1 and Group 2 as follows: in Group 1 (Figure 5A), 92% of healthy vaccinated children were responders; in Group 2 (Figure 5B), 100% of those infected and vaccinated were found to be responders. In Group 3 (infected, non-vaccinated children), 20% of the children had antibodies specific to the Sm14 protein, but even if their responses were relatively weak, as shown in Figure 5C, it can be said that a natural response in the infected children existed. The majority isotype in the vaccinated children was IgG1. IgG3 was also induced by the vaccine but at a lower proportion. This was also observed in the non-vaccinated infected children. Finally, the production of IgG4 was observed only in infected children and not in healthy vaccinated children.

#### 3.2.3. Cell-Mediated Immunity

Evaluating the main PBMC subpopulations of the volunteer children participating in the Phase IIb vaccine trial in Senegal, we observed increased percentages of total T-cell populations (CD3^+^) in the controls (Group 3) when comparing data from the baseline and Day 148. In contrast, a decrease was detected in Group 2 when comparing the baseline data with those on Day 84 (Table 6). A reduction was also observed in the absolute numbers of CD8^+^ (CD3^+^CD8^+^) T-cell subpopulation in Group 1 when comparing the baseline with Day 84 (Table 6). No differences were observed in the percentages of CD4^+^ (CD3^+^CD4^+^) and CD8^+^ T-cell subpopulations or in the absolute numbers of total T-cell populations and CD4^+^ T-cell subpopulations (Table 6). With regard to the B-cell population (CD3^−^CD19^+^), we detected a decrease in the percentage of these cells, both in the control (Group 3) and in Group 2 when comparing baseline data with those recorded on Day 84 and Day 148. Meanwhile, an increase was verified in Group 1 when comparing the baseline with Day 84. No differences in absolute numbers were detected (Table 6).

Conversely, we verified an elevated percentage of monocytes (CD3^−^CD14^+^) in the controls (Group 3) when comparing baseline data with those on Day 148 and in Group 2 when comparing baseline data with those on Day 84 and Day 148. The increase detected in the percentages of Group 2 was also identified in absolute numbers (Table 6).

In addition, we looked for the CD4^+^ and CD8^+^ memory T cell profile of these children after vaccination. Surprisingly, we observed an increase in naïve (CCR7^+^CD45RA^+^) CD4^+^ and CD8^+^ T cell populations in Group 1 (healthy, vaccinated children) when comparing baseline data with data from Day 84 and Day 148. The controls (Group 3) also presented an elevated percentage of CD4 naïve T cells when comparing baseline data with those recorded on Day 148 (Table 7). In contrast, we detected diminished percentages of effector CD4^+^ and CD8^+^ T cells (CCR7^−^CD45RA^+^) on Day 84 and on Day 148, respectively, in Group 1 compared with the baseline (Table 7 and Table 8). Regarding effector memory T cells (CCR7^−^CD45RA^−^), we found a decrease in CD4^+^ T cells in Group 1 when comparing the baseline data with those on Day 84 and Day 148 (Table 7) and in CD8^+^ T cells in Group 2 and in Group 1 when comparing the baseline data with day data (Table 8). In Group 2, this reduction was abrogated on Day 148. In respect to central memory T cells (CCR7^+^CD45RA^−^), no differences were identified in CD4^+^ T cells (Table 7). However, a decline was verified in the CD8^+^ T cell subpopulation in Group 2 when comparing the baseline data with Day 84 data (Table 8).

We also attempted to evaluate if CD4^+^ and CD8^+^ T cells from the vaccinated children were able to produce IFN-γ or TNF-α when in contact with rSm14. PBMC samples were then activated with non-specific PMA + ionomycin (used as control of cell activation) and specific (rSm14) stimuli for 4 h. After stimulation, PBMCs were stained for intracellular IFN-γ and TNF-α, and cytokine production was detected by flow cytometry. PBMC activation with PMA and ionomycin induced IFN-γ and TNF-α production by CD4^+^ and CD8^+^ T cells from all three groups on baseline, Day 84, and Day 148, indicating that cellular activation was successful.

**Figure 6:**
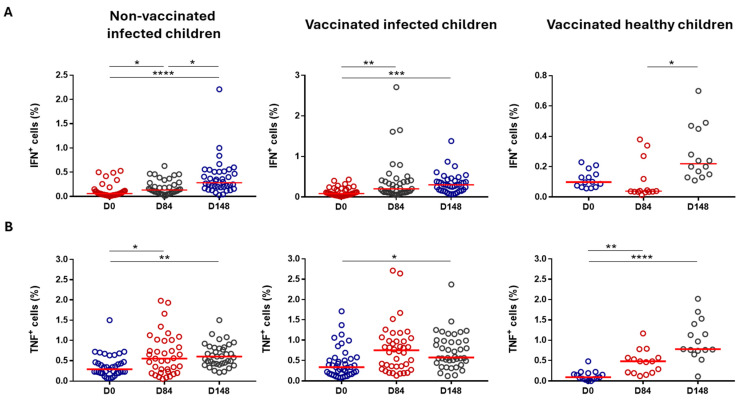
Cytokine production by CD4^+^ T cells from the vaccinated Senegalese children (Phase IIb). Each dot represents an individual value, with the horizontal lines signifying median values. (**A**) IFN-γ production by rSm14-activated CD4^+^ T cells on Day 0, Day 84, and Day 148; (**B**) TNF-α production by rSm14-activated CD4^+^ T cells on Day 0, Day 84, and Day 148; * *p* < 0.05; ** *p* < 0.01; *** *p* < 0.001; **** *p* < 0.0001.

After activation with the antigen-specific stimulus (rSm14), we detected an increase in both IFN-γ and TNF-α production by CD4^+^ T cells (Figure 6A and Figure 6B, respectively). Regarding IFN-γ production, this enhancement was verified in the control (Group 3) and in Group 2 when comparing baseline data with those from Day 84 and Day 148. In these groups, increased levels of IFN-γ-producing CD4^+^ T cells were higher on Day 148 compared with the baseline. We also observed an increase in Group 1 when comparing data on Day 148 and Day 84 (Figure 6A). However, the same differences were found also in non-activated cells. In fact, we even detected elevated levels of IFN-γ in Group 1 when comparing the baseline data with those recorded on Day 148. Differences between IFN-γ-producing non-activated and rSm14-activated CD4 T cells were detected only at the baseline in Group 1.

Meanwhile, TNF-α production by CD4^+^ T cells was elevated in the rSm14-activated cells in the controls (Group 3) and in Group 1 when comparing baseline data with those on Day 84 and Day 148 and in Group 3 when comparing baseline data and Day 148 data (Figure 6B). Analysing the non-activated CD4^+^ T cells, the same differences in the percentage of TNF-α producing cells were found in Group 1. Furthermore, an increased TNF-α production by these cells was observed in Group 3 when comparing baseline data with those on Day 84. In contrast, we observed a decrease in the controls (Group 3) at baseline and in Group 1 on Day 148 when comparing TNF-α production between non-activated and rSm14-activated CD4^+^ T cells.

**Figure 7:**
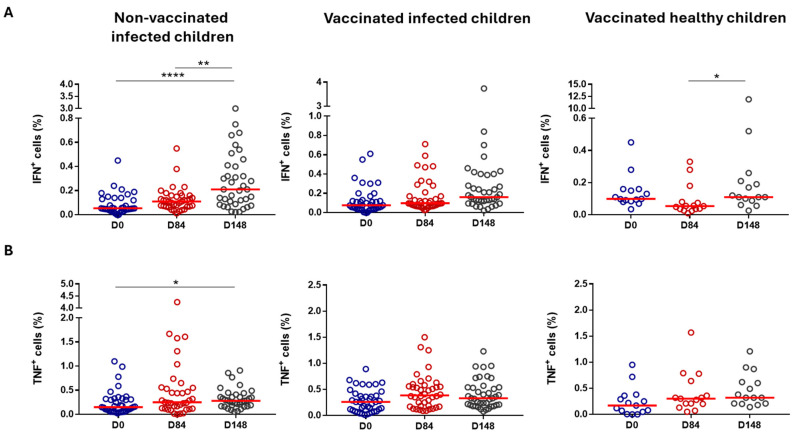
Cytokine production by CD8^+^ T cells from vaccinated children (Phase IIb trial). Each dot represents an individual value, with the horizontal lines signifying median values. (**A**) IFN-γ production by rSm14-activated CD8^+^ T cells on Day 0, Day 84, and Day 148; (**B**) TNF-α production by rSm14-activated CD8^+^ T cells on Day 0, Day 84, and Day 148; * *p* < 0.05; ** *p* < 0.01; **** *p* < 0.0001.

When evaluating cytokine production by CD8^+^ T cells, we also observed increased production of both IFN-γ and TNF-α after cell stimulation with rSm14 (Figure 7A and Figure 7B, respectively). Elevated IFN-γ-producing CD8^+^ T cells were detected in the rSm14-activated cells from the controls (Group 3) and from Group 1 when comparing Day 148 to the baseline and to Day 84 (Figure 7A). This increased production was also verified in non-activated cells from the same groups and from vaccinated infected children. In the non-activated cells, an enhanced percentage of IFN-γ-producing CD8^+^ T cells from the controls (Group3) were noticed even when comparing Day 84 to the baseline data. Activated (by rSm14) and non-activated CD8^+^ T cells from Group 1 presented a non-significant decrease in IFN-γ production on Day 84 that was abrogated on Day 148, returning to basal levels from the baseline (Figure 6A. The enhanced IFN-γ-producing CD8^+^ T-cell population was verified in rSm14-activated cells when compared with non-activated cells in the controls (Group 3) at the baseline and on Day 84, and in Group 2 at the baseline.

The percentage of TNF-α-producing CD8^+^ T cells was increased in the rSm14-activated cells in the controls (Group 3) on Day 148 when compared with the baseline (Figure 7B). However, an elevated percentage of these cells was also found in the non-activated cells. Enhanced percentages were detected in the controls (Group 3) when comparing Day 84 to the baseline and in Group 1 when comparing Day 148 to the baseline. Nevertheless, we observed that rSm14-activated cells presented fewer CD8^+^ T cells capable of producing TNF-α than the non-activated cells. This fact was evident in the controls (Group 3) at the baseline, on Day 84, and on Day 148 and in Group 2 on Day 84. In contrast, CD8^+^ T cells from Group 2 were able to produce more TNF-α at the baseline after rSm14 activation than the non-activated cells.

Vaccine-mediated protection can occur due to the generation of humoral responses, essentially antibodies produced by B cells, and/or cellular responses, basically mediated by CD4 or CD8 T cells [29]. T-cell modulation was punctually verified in the vaccinated groups of school children. A decrease in the percentage of T cells was observed in Group 2 (previously infected, vaccinated children) and in the absolute numbers of CD8^+^ T cells in Group 1 (healthy, vaccinated children). However, both decreases were detected on Day 84 and were not sustained until Day 148.

Our data also showed that monocytes were also modulated, as increased percentages of these cells were detected in Group 2 (previously infected, vaccinated children) and in Group 3 (previously infected, non-vaccinated children). This increase seems to be related to schistosomiasis infection, since it occurred only in children from previously infected groups, vaccinated or not. Regarding B cells, a decrease was verified in Group 2 (previously infected, vaccinated children) and in Group 3 (previously infected, non-vaccinated children), while an increase was detected in Group 1 (healthy, vaccinated children).

Our data showed decreased percentages of effector memory CD4 and CD8 T cells in Group 1 (healthy, vaccinated children) and in both effector and central memory CD8 T cells in Group 2 (previously infected, vaccinated children). Surprisingly, we detected an increase in naïve CD4 and CD8 T cells and a decrease in effector CD4 and CD8 T cells in Group 1 (healthy, vaccinated children). Although we did not observe an increase in effector and central memory T cells in children who received the Sm14+GLA-SE vaccine, we did not evaluate antigen-specific T cells. Therefore, we may be missing this data as we look at a total pool of central and effector memory cells. Thus, the ability of the vaccine Sm14+GLA-SE to generate effector and central memory T cells should not be discarded, and it would be interesting to evaluate antigen-specific memory T cells.

During the Sm14+GLA-SE vaccine Phase II clinical trial in school children, cytokine production analysis was evaluated by flow cytometry and by luminex. Flow cytometry data revealed a decrease in the percentages of TNF-α-producing CD4 and CD8 T cells after rSm14-specific stimulation. The decrease observed in CD4 T-cell subpopulations was detected only in Group 1 (healthy, vaccinated children). We did not detect a modulation of IFN-γ production. However, differently from what was done during Phase I of the clinical trial, we did not use costimulatory molecules during PBMC stimulation with rSm14 [13]. The presence of costimulatory molecules during stimulation might have been fundamental for IFN-γ production. The absence of these molecules may also explain the slight increase detected in cytokine production during Phase 2b of the clinical trial when compared with Phase 1 of the clinical study.

## 4. Discussion

Despite the extensive use of chemotherapy distributed by MDA and well-functioning delivery networks, the prevalence and transmission rates of schistosomiasis have not significantly declined (in fact, they have increased in some places) over the past 30 years. For this reason, we strongly question the widely accepted preventive chemotherapy strategy. The recently published Vaccine Value Profile for Schistosomiasis [30] advocates a multi-faceted control programme. This approach would offer several significant advantages, particularly if it included a vaccine, as suggested by Bergquist et al. [31]. For example, even a partially protective vaccine would reduce the incidence of infection, lowering overall disease burdens and improving public health outcomes. Importantly, a vaccine should complement massive chemotherapy by providing a sustainable, long-term solution that would eventually lead to the elimination of schistosomiasis, as envisioned in WHO’s Road Map and the United Nation (UN)’s Sustainable Development Goals (SDGs). In addition, by reducing the prevalence of this disease, the economic burden in the endemic countries would be alleviated and would lead to lower healthcare costs.

Since schistosomiasis affects impoverished, rural populations more than others, a vaccination scheme would help close disparity gaps and assist people without access to safe, clean water. Children are particularly vulnerable to schistosomiasis, as long-term high worm burdens in this age group cause cognitive impairment and stunted growth. If given early in life, vaccination would contribute to a better life for children and ensure better health outcomes in the endemic areas. If a significant portion of vulnerable populations around the world, particularly in sub-Saharan Africa, could be vaccinated, transmission of the disease would be reduced. Public health programmes that regularly treat all children with deworming drugs, including praziquantel, do not appear to improve height, haemoglobin levels, cognition, school performance, or mortality. Although historical studies carried out decades ago reported significant effects on weight gain, more recent, larger studies have not confirmed this [32].

The Global Vaccine Action Plan (GVAP) defines immunization as a human right and a community and governmental responsibility. GVAP argues that the 20th century was the century of treatment based on the development of antibiotic drugs, and that the 21st century should be the century of vaccines with the potential to eradicate, eliminate, or control a number of serious, life-threatening, or debilitating infectious diseases, with immunization at the core of preventive strategies [33].

The Oswaldo Cruz Foundation in Brazil developed a recombinant anti-schistosomiasis vaccine based on the Sm14 protein formulated with the new-generation adjuvant GLA-SE from the Access to Advanced Health Institute (AAHI) in Seattle in the US. This paper reports advanced clinical trials conducted in adults (Phase IIa) and school children (Phase IIb) in Africa, demonstrating excellent safety and robust immunogenicity in endemic areas for *S. mansoni* and *S. haematobium*. In these trials, both adults and school children developed a high antigen-specific immune response following vaccination. Total IgG levels were elevated in the vaccinated groups, whereas they remained stable, without change in the non-vaccinated group. The healthy children exhibited a steeper elevation in IgG levels after receiving the vaccines compared with those who had been infected (and cured) previously. Additionally, the percentage of responders was higher in healthy vaccinated children than in their counterparts who had experienced previous infection(s). Also, the cell-mediated immune response was activated, as seen by the levels of cytokines produced upon stimulation of T cells with Sm14.

It is interesting to speculate on why the group of healthy children—those without prior schistosomiasis infection—exhibited a better response to vaccination in terms of both antibody titres and cellular immune response. While this is not a specific answer, one possibility is that healthier, uninfected children are capable of developing a more robust and effective immune response precisely due to their lack of prior infection. The absence of chronic parasitic exposure may allow their immune system to mount a stronger and more efficient response to the vaccine.

Another factor to consider is that chronic parasitic infections such as schistosomiasis, are known to induce regulatory immune responses that may dampen the effectiveness of vaccines. Infected children may have an altered immune environment, where the immune system is constantly engaged in modulating responses to persistent parasitic antigens. This could lead to a less vigorous reaction to vaccination when compared with children who have never been exposed to the parasite. Therefore, differences in immune system activation and regulation between infected and uninfected children could be a key explanation for the observed disparity in vaccine response.

The Immunization Agenda 2030 (IA2030) sets a bold global vision and strategy for vaccines: to leave no one behind. Lessons learned from GVAP and its implementation can help shape IA2030. The COVID-19 pandemic, which impacted the world and accelerated progress in vaccinology, highlighted the need for comprehensive vaccine accessibility. To leave no one behind also entails the development of new vaccines targeting the parasites prevalent in low- and middle-income countries. The Sm14 candidate anti-schistosomiasis vaccine, developed and manufactured as a humanitarian vaccine by an endemic country in the Global South, aims to collaborate and serve other endemic regions in a South–South cooperation. We believe initiatives like Sm14 should be prioritized strategically to reduce inequalities and enhance well-being, aligning with the UN SDGs.

## 5. Conclusions

Sm14 was safe, with no serious adverse events and only minor events in a few patients, both adults and children. The vaccine induced strong antibody and cytokine responses in endemic areas for *S. mansoni* and *S. haematobium*. In these trials, both adults and school children developed a high antigen-specific immune response following vaccination. Overall, this study advances the field toward a vaccine for schistosomiasis.

## Figures and Tables

**Table 1:** Characteristics of school children participating in the Phase IIb clinical trial.

Trial Characteristic		Healthy, Vaccinated Children (Group 1)	Previously Infected, Vaccinated Children (Group 2)	Previously Infected, Non-Vaccinated Children (Group 3)
Sample size		15	40	40
Age				
Years	Mean (SD)	9.3 (1.2)	9.4 (1.1)	9.2 (1.1)
Median	(min, max)	9 (8, 11)	9.5 (8, 11)	9.0 (8, 11)
Sex %	males (n)	40.0 (6)	50.0 (20)	42.5 (17)
Infection type				
*S. haematobium* only	% (n)	0 (0)	50.0 (20)	62.5 (25)
*S. mansoni* only	% (n)	0 (0)	35.0 (14)	27.5 (11)
Mixed infection	% (n)	0 (0)	15.0 (6)	10.0 (4)

SD = standard deviation; Group 3 is the control group.

**Table 2:** Schematic temporal outline of the inclusion and vaccination of school-age children (Phase IIb).

Healthy group: first vaccine injection (D0)							
**Week 1**							
	M	T	W	T	F	Sat	Sun
Group 1 (Healthy children + rSm14)	1		2		3		
Group 2 (Previously infected children + rSm14)							
Group 3 (Unvaccinated, previously infected children)							
Inclusion	Week 1 = 6 subjects						
Vaccination	Week 1 = 6 subjects						
**Week 2**							
	M	T	W	T	F	Sat	Sun
Group 1 (Healthy children + rSm14)	3		3		3		
Group 2 (Previously infected children + rSm14)							
Group 3 (Unvaccinated, previously infected children)							
Inclusion	Week 2 = 9 subjects						
Vaccination	Week 2 = 9 subjects						
**Infected group: first vaccine injection (D0)**							
**Week 3**							
	M	T	W	T	F	Sat	Sun
Group 1 (Healthy children + rSm14)							
Group 2 (Previously infected children + rSm14)	1		2		3		
Group 3 (Unvaccinated, previously infected children)	1		2		3		
Inclusions	Week 3 = 12 subjects						
Vaccination	Week 3 = 6 subjects						
**Week 4**							
	M	T	W	T	F	Sat	Sun
Group 1 (Healthy children + rSm14)							
Group 2 (Previously infected children + rSm14)	3		3		3		
Group 3 (Unvaccinated, previously infected children)	3		3		3		
Inclusion	Week 4 = 18 subjects						
Vaccination	Week 4 = 9 subjects						
**Week 5**							
	M	T	W	T	F	Sat	Sun
Group 1 (Healthy children + rSm14)							
Group 2 (Previously infected children + rSm14)	3		3		3		
Group 3 (Unvaccinated, previously infected children)	3		3		3		
Inclusion	Week 5 = 18 subjects						
Vaccination	Week 5 = 9 subjects						
**Week 6**							
	M	T	W	T	F	Sat	Sun
Group 1 (Healthy children + rSm14)							
Group 2 (Previously infected children + rSm14)	3		3		3		
Group 3 (Unvaccinated, previously infected children)	3		3		3		
Inclusion	Week 6 = 18 subjects						
Vaccination	Week 6 = 9 subjects						
**Week 7**							
	M	T	W	T	F	Sat	Sun
Group 1 (Healthy children + rSm14)							
Group 2 (Previously infected children + rSm14)	3		3		1		
Group 3 (Unvaccinated, previously infected children)	3		3		1		
Inclusion	Week 7 = 14 subjects						
Vaccination	Week 7 = 7 subjects						

Group 1 = 15 healthy children vaccinated with rSm14; Group 2 = 40 infected children vaccinated with rSm14; Group 3 (control) = 40 unvaccinated infected children.

**Table 3:** Clinical observations in the adults (n = 15) during the first 48 h after administration of 50 µg rSm14 with different amounts of the GLA-SE adjuvant.

Adverse Events	GLA-SE Content	AE After First Injection * (95% CI)	AE After Second Injection ** (95% CI)	AE After Third Injection *** (95% CI)
Serious	2.5 μg	0.00 (0.00, 0.22)	0.00 (0.00, 0.22)	0.00 (0.00, 0.22)
	5.0 μg	0.00 (0.00, 0.22)	0.00 (0.00, 0.22)	0.00 (0.00, 0.22)
Pain at site of injection	2.5 μg	0.13 (0.02, 0.40)	0.00 (0.00, 0.22)	0.07 (0.002, 0.32)
	5.0 μg	0.20 (0.04, 0.48)	0.20 (0.04, 0.48)	0.13 (0.02, 0.40)
Heavy arm post injection	2.5 μg	0.00 (0.00, 0.22)	0.20 (0.04, 0.48)	0.07 (0.002, 0.32)
	5.0 μg	0.07 (0.002, 0.32)	0.27 (0.08, 0.55)	0.20 (0.04, 0.48)
Pruritus	2.5 μg	0.00 (0.00, 0.22)	0.00 (0.00, 0.22)	0.13 (0.02, 0.40)
	5.0 μg	0.00 (0.00, 0.22)	0.00 (0.00, 0.22)	0.00 (0.00, 0.22)
Dizziness and/or headache	2.5 μg	0.00 (0.00, 0.22)	0.00 (0.00, 0.22)	0.00 (0.00, 0.22)
	5.0 μg	0.00 (0.00, 0.22)	0.07 (0.002, 0.32)	0.00 (0.00, 0.22)
Acute gastroenteritis	2.5 μg	0.00 (0.00, 0.22)	0.00 (0.00, 0.22)	0.07 (0.002, 0.32)
	5.0 μg	0.00 (0.00, 0.22)	0.00 (0.00, 0.22)	0.00 (0.00, 0.22)

AE = adverse event; CI = confidence interval; * given from Day 0 to Day 2; ** given from Day 28 to Day 30; *** given from Day 56 to Day 58. Numbers shaded in grey indicate the presence of at least one AE.

**Table 4:** Clinical observations in school-age children (n = 95) during the first 48 h after vaccination.

Adverse Event (AE)	Group	No. ^a^	AE After First Injection ^b^ Event (95% CI)	No. ^a^	AE After Second Injection ^c^ Event (95% CI)	No. ^a^	AE After Third Injection ^d^ Event (95% CI)
Serious (Grades 3–4)	Healthy	0/15	0.00 (0.00, 0.22)	0/15	0.00 (0.00, 0.22)	0/15	0.00 (0.00, 0.22)
	Pre-infected	0/40	0.00 (0.00, 0.09)	0/40	0.00 (0.00, 0.09)	0/40	0.00 (0.00, 0.09)
Pain at site of infection	Healthy	0/15	0.00 (0.00, 0.22)	2/15	0.13 (0.02, 0.40)	0/15	0.00 (0.00, 0.22)
	Pre-infected	2/40	0.05 (0.01, 0.17)	3/40	0.08 (0.02, 0.20)	3/40	0.08 (0.02, 0.20)
Heavy arm post inject.	Healthy	0/15	0.00 (0.00, 0.22)	0/15	0.00 (0.00, 0.22)	0/15	0.00 (0.00, 0.22)
	Pre-infected	0/40	0.00 (0.00, 0.09)	0/40	0.00 (0.00, 0.09)	1/40	0.02 (0.001, 0.13)
Pruritus	Healthy	1/15	0.00 (0.00, 0.22)	1/15	0.00 (0.00, 0.22)	1/15	0.00 (0.00, 0.22)
	Pre-infected	0/40	0.00 (0.00, 0.09)	0/40	0.00 (0.00, 0.09)	1/40	0.02 (0.001, 0.13)
Swelling	Healthy	0/15	0.00 (0.00, 0.22)	0/15	0.00 (0.00, 0.22)	0/15	0.00 (0.00, 0.22)
	Pre-infected	0/40	0.00 (0.00, 0.09)	0/40	0.00 (0.00, 0.09)	1/40	0.02 (0.001, 0.13)
Fever	Healthy	1/15	0.07 (0.002, 0.32)	0/15	0.00 (0.00, 0.22)	0/15	0.00 (0.00, 0.22)
	Pre-infected	0/40	0.00 (0.00, 0.09)	1/40	0.02 (0.001, 0.13)	1/40	0.02 (0.001, 0.13)
Headache	Healthy	1/15	0.07 (0.002, 0.32)	0/15	0.00 (0.00, 0.22)	0/15	0.00 (0.00, 0.22)
	Pre-infected	1/40	0.02 (0.001, 0.13)	2/40	0.05 (0.01, 0.17)	0/40	0.00 (0.00, 0.09)
Abdominal pain	Healthy	0/15	0.00 (0.00, 0.22)	0/15	0.00 (0.00, 0.22)	0/15	0.00 (0.00, 0.22)
	Pre-infected	1/40	0.02 (0.001, 0.13)	0/40	0.00 (0.00, 0.09)	0/40	0.00 (0.00, 0.09)
Vomiting	Healthy	0/15	0.00 (0.00, 0.22)	1/15	0.07 (0.002, 0.32)	0/15	0.00 (0.00, 0.22)
	Pre-infected	1/40	0.02 (0.001, 0.13)	0/40	0.00 (0.00, 0.09)	0/40	0.00 (0.00, 0.09)

^a^ AE level presented as number out of all vaccinated (no./all); ^b^ occurring between Day 0 and Day 2; ^c^ occurring between Day 28 and Day 30; ^d^ occurring between Day 56 and Day 58; CI = confidence interval; Pre-infected = infected at selection and treated with praziquantel before inclusion; areas shaded in grey highlight presence of at least one AE.

**Table 5:** IgG levels over time and classification in the clinical trial in school children (Phase IIb trial).

Blood Collection (Day)		Group			
	IgG Response	Healthy, Vaccinated ^a^ Group 1	Previously Infected, Vaccinated ^b^ Group 2	Previously Infected, Non-Vaccinated ^c^ Group 3	*p*-Value ^d^
0	Mean (SD)	56 (176)	321 (627)	324 (751)	
	Median (min, max)	0 (0, 688)	2 (0, 2306)	0 (0, 3370)	
	Responder % (n)	6.7% (1)	25.0% (10)	20.0% (8)	0.40
84	Mean (SD)	3533 (1778)	2555 (1672)	262 (591)	
	Median (min, max)	3118 (1763, 9180)	2211 (22, 7326)	0 (0, 2332)	
	Responder % (n)	100 (15)	92.5 (37)	20.0 (8)	<0.001
148	Mean (SD)	2078 (1210)	1626 (1177)	231 (536)	
	Median (min, max)	1620 (920, 5624)	1605 (1, 4998)	0 (0, 2072)	
	Responder % (n)	100 (15)	90.0 (36)	20.0 (8)	<0.001
400+ ^e^	Mean (SD)	1322 (973)	1205 (1014)	317 (604)	
	Median (min, max)	1203 (61, 3476)	1110 (0, 3336)	1 (0, 2454)	
	Responder % (n)	92.9 (13)	73.7 (28)	33.3 (13)	<0.001

^a^ n = 15; ^b^ n = 40; ^c^ n = 40; ^d^ Fisher exact test of association between responder (defined as having IgG > 150) and number in the group; ^e^ there were some missing values at this point in time: one in Group 1, two in Group 2, and one in Group 3.

**Table 6:** Peripheral blood cells after vaccination in Senegalese school children (Phase IIb).

Cell Population	Healthy, Vaccinated Children (Group 1)		Previously Infected, Vaccinated Children (Group 2)		Previously Infected, Non-Vaccinated Children (Group 3)	
T cells (CD3^+^)	Not significant		↓ Day 84 (%)	*p* = 0.0036	↑ Day 148 (%)	*p* = 0.0349
CD4^+^ T cells	Not significant		Not significant		Not significant	
CD8^+^ T cells	↓ Day 84 (AN)	*p* = 0.0105	Not significant		Not significant	
B cells	↑ Day 84 (%)	*p* = 0.0105	↓ Day 84 (%)	*p* = 0.0003	↓ Day 148 (%)	*p* = 0.0017
Monocytes	Not significant		↑ Day 84/Day 148 (%/AN)	*p* = 0.0029/0.0063 *p* = 0.0110/0.0417	↑ Day 148 (%)	*p* = 0.0250

Arrows indicate statistically significant change and direction in the level of value compared with Day 0; AN = absolute number.

**Table 7:** Naïve, effector, and memory CD4 T cells in peripheral blood after vaccination in Senegalese school children (Phase IIb trial).

CD4 T-Cell Subpopulation	Healthy, Vaccinated Children (Group 1)		Previously Infected, Vaccinated Children (Group 2)	Previously Infected, Non-Vaccinated Children (Group 3)	
Naïve T cells	↑ Day 84	*p* = 0.0016	Not significant	↑ Day 148	*p* = 0.0250
	↑ Day 148	*p* < 0.0001			
Effector T cells	↓ Day 84	*p* = 0.0030	Not significant	Not significant	
	↓ Day 148	*p* = 0.0030			
EMT cells	↓ Day 84	*p* < 0.0001	Not significant	Not significant	
	↓ Day 148	*p* = 0.0105			
CMT cells	Not significant		Not significant	Not significant	

Arrows indicate statistically significant change and direction in the level of value compared with Day 0; AN = absolute number. EMT cells = effector memory T cells; CMT cells = central memory T cells.

**Table 8:** Naïve, effector, and memory CD8 T cells in peripheral blood after vaccination in Senegalese school children (Phase IIb trial).

CD8 T-Cell Subpopulation	Healthy, Vaccinated Children (Group 1)		Previously Infected, Vaccinated Children (Group 2)		Previously Infected, Non-Vaccinated Children (Group 3)
Naïve T cells	↑ Day 84	*p* = 0.0001	Not significant		Not significant
	↑ Day 148	*p = 0.0411*			
Effector T cells	↓ Day 148	*p* = 0.0185	Not significant		Not significant
EMT cells	↓ Day 84	*p* = 0.0008	↓ Day 84	*p* = 0.0052	Not significant
CMT cells	Not significant		↓ Day 84	*p* = 0.0024	Not significant

Arrows indicate statistically significant change and direction in the level of value compared with Day 0; AN = absolute number. EMT cells = effector memory T cells; CMT cells = central memory T cells.

## Data Availability

All data that were analysed during this study are included in this published article.

## Supplementary Materials

The following supporting information can be downloaded at: https://www.mdpi.com/article/10.3390/vaccines13030316/s1, Figure S1: The Study Area: Lower Senegal River Basin, Northern Senegal. Figure S2: Gate strategy for PBMC phenotypic analysis from children who receive the Sm14+GLA-SE vaccine. Table S1: Laboratory tests applied in all individuals. Table S2: Panels of fluorescent monoclonal antibodies for phenotypic analysis of peripheral blood mononuclear cells. Table S3: Mononuclear cell subpopulations analysed by flow cytometry and with their corresponding monoclonal antibody defined membrane markers. Table S4: Antibodies, isotype controls and dye used in flow cytometry. Table S5: Immunoglobulin responses to Sm-14: total IgG and subclasses IgG1, IgG2, IgG3, IgG4, and IgGE—proportion (95% CI) of individuals with fold-rise change ≥ 4 per time and group.
