# Supplementary material for: The Sm14+GLA-SE Recombinant Vaccine Against Schistosoma mansoni and S. haematobium in Adults and School Children: Phase II Clinical Trials in West Africa
Source: Vaccines (Basel). 2025 Mar 16;13(3):316. doi: 10.3390/vaccines13030316 (PMC11946331; doi:10.3390/vaccines13030316)
Supplement: Supplementary file 1 [file vaccines-13-00316-s001.zip › Table S1 (reviewed) vaccines-3455148.pdf]

Supplement Table S1. Laboratory tests applied in all individuals.

Haematology	Liver biochemistry	Kidney biochemistry
**C-reactive protein***	Aspartate aminotransferase (AST)*	Creatinine*
**Haemoglobin****	alanine aminotransferase (ALT)*	Urea*
**Haematocrit****	Total bilirubin*	Proteinemia*
**Erythrocytes****	Free bilirubin*	Albuminemia*
**Leukocytes****	Conjugated bilirubin*	Proteinuria***
**Neutrophils****		Albuminuria***
**Eosinophils****		
**Basophils****		
**Lymphocytes****		
**Monocytes****		
**Platelets****		

*Dry tube; ** EDTA tube; ***urine test by test strips.

Although most eosinophil counts were above the normal range of laboratory standards, they were not associated with any other abnormal laboratory findings or clinical symptoms that would indicate a possible relationship to the vaccine. Furthermore, many elevated values were already present at baseline, indicating that the participant had these values before vaccination.
