# Supplementary material for: The Sm14+GLA-SE Recombinant Vaccine Against Schistosoma mansoni and S. haematobium in Adults and School Children: Phase II Clinical Trials in West Africa
Source: Vaccines (Basel). 2025 Mar 16;13(3):316. doi: 10.3390/vaccines13030316 (PMC11946331; doi:10.3390/vaccines13030316)
Supplement: Supplementary file 1 [file vaccines-13-00316-s001.zip › Table S2.pdf]

Supplement Table S2. Panels of fluorescent monoclonal antibodies for phenotypic analysis of peripheral blood mononuclear cells.

Fluorochromes	Antibody panel 1	Antibody panel 2	Antibody panel 3	Antibody panel 4	Antibody panel 5
	**Activation**	**Memory**	**Cytokines**	**Subpopu-lations**	**Cytokines controls**
**FITC**	Viability	Viability	Viability	Viability	Viability
**PE**	CD49d	CCR7	IFN-γ	CD19	mouse IgG1
**BB700**	CD8	CD8	CD8	CD8	CD8
**PE-Cy7**	HLA-DR	CD45RA	TNF-α	CD14	mouse IgG1
**APC**	CD3	CD3	CD3	CD3	CD3
**APC-Cy7**	CD4	CD4	CD4	CD4	CD4
