# Supplementary material for: The Sm14+GLA-SE Recombinant Vaccine Against Schistosoma mansoni and S. haematobium in Adults and School Children: Phase II Clinical Trials in West Africa
Source: Vaccines (Basel). 2025 Mar 16;13(3):316. doi: 10.3390/vaccines13030316 (PMC11946331; doi:10.3390/vaccines13030316)
Supplement: Supplementary file 1 [file vaccines-13-00316-s001.zip › Table S3.pdf]

Supplement Table S3. Mononuclear cell subpopulations analysed by flow cytometry and with their corresponding monoclonal antibody defined membrane markers.

Leukocyte Subpopulations	Combination of Membrane Markers
**CD4 T Cell**	$CD3^{+}/CD4^{+}/CD8^{-}$
**CD8 T Cell**	$CD3^{+}/CD4^{-}/CD8^{+}$
**Activated CD4 T Cell**	$CD3^{+}/CD4^{+}/CD8^{-}/HLA\text{-}DR^{+}/CD49d^{+}$
**Activated CD8 T Cell**	$CD3^{+}/CD4^{-}/CD8^{+}/HLA\text{-}DR^{+}/CD49d^{+}$
**Central Memory CD4 T Cell**	$CD3^{+}/CD4^{+}/CD8^{-}/CCR7^{+}/CD45RA^{-}$
**Effector Memory CD4 T Cell**	$CD3^{+}/CD4^{+/}CD8^{-}/CCR7^{-}/CD45RA^{-}$
**Effector CD4 T Cell**	$CD3^{+}/CD4^{+}/CD8^{-}/CCR7^{-}/CD45RA^{+}$
**Naive CD4 T Cell**	$CD3^{+}/CD4^{+}/CD8^{-}/CCR7^{+}/CD45RA^{+}$
**Central Memory CD8 T Cell**	$CD3^{+}/CD4^{-}/CD8^{+}/CCR7^{+}/CD45RA^{-}$
**Effector Memory CD8 T Cell**	$CD3^{+}/CD4^{-}/CD8^{+}/CCR7^{-}/CD45RA^{-}$
**Effector CD8 T Cell**	$CD3^{+}/CD4^{-}/CD8^{+}/CCR7^{-}/CD45RA^{+}$
**Naive CD8 T Cell**	$CD3^{+}/CD4^{-}/CD8^{+}/CCR7^{+}/CD45RA^{+}$
**IFN-γ producing CD4 T cells**	$CD3^{+}/CD4^{+}/CD8^{-}/IFN\text{-}\gamma^{+}/TNF\text{-}\alpha^{-}$
**TNF-α producing CD4 T Cells**	$CD3^{+}/CD4^{+}/CD8^{-}/IFN\text{-}\gamma^{-}/TNF\text{-}\alpha^{+}$
**IFN-γ producing CD8 T Cells**	$CD3^{+}/CD4^{-}/CD8^{+}/IFN\text{-}\gamma^{+}/TNF\text{-}\alpha^{-}$
**TNF-α producing CD8 T Cells**	$CD3^{+}/CD4^{-}/CD8^{+}/IFN\text{-}\gamma^{-}/TNF\text{-}\alpha^{+}$
**B Cells**	$CD3^{-}/CD19^{+}$
**Monocytes**	$CD3^{-}/CD14^{+}$
