# Supplementary material for: The Sm14+GLA-SE Recombinant Vaccine Against Schistosoma mansoni and S. haematobium in Adults and School Children: Phase II Clinical Trials in West Africa
Source: Vaccines (Basel). 2025 Mar 16;13(3):316. doi: 10.3390/vaccines13030316 (PMC11946331; doi:10.3390/vaccines13030316)
Supplement: Supplementary file 1 [file vaccines-13-00316-s001.zip › Table S4.pdf]

Supplement Table S4. Antibodies, isotype controls and dye used in flow cytometry*.

Antibody, Isotype control or Dye	Catalognumber	Dilution
APC mouse anti-human CD3	555335	1:20
APC-H7 mouse anti-human CD4	560158	1:40
BB700 mouse anti-human CD8	566452	1:5,120
PE mouse anti-human CD19	555413	1:10
FITC mouse anti-human CD19	555412	1:10
PE-CY™7 mouse anti-human CD14	557742	1:320
PE-CY™7 mouse anti-human CD45RA	560675	1:160
PE mouse anti-human CD49d	555503	1:20
PE mouse anti-human CD197 (CCR7)	560765	1:5
PE-CY™7 mouse anti-human HLA-DR	560651	1:10
PE mouse anti-human IFN-γ	561056	1:10
PE-CY™7 mouse anti-human TNF-α	557647	1:20
PE mouse IgG1, κ Isotype Control	554680	1:10
PE-CY™7 mouse IgG, κ Isotype Control	557646	1:20
Fixable Viability Stain 520 (FVS520)	564407	1:1,000

*All reagents were produced by BD Biosciences,Franklin Lakes, NJ, USA
