# Supplementary material for: The Sm14+GLA-SE Recombinant Vaccine Against Schistosoma mansoni and S. haematobium in Adults and School Children: Phase II Clinical Trials in West Africa
Source: Vaccines (Basel). 2025 Mar 16;13(3):316. doi: 10.3390/vaccines13030316 (PMC11946331; doi:10.3390/vaccines13030316)
Supplement: Supplementary file 1 [file vaccines-13-00316-s001.zip › Table S5.pdf]

**Supplement Table S5.** Immunoglobulin responses to Sm-14: total IgG and subclasses IgG1, IgG2, IgG3, IgG4, and IgGE—proportion (95% CI) of individuals with fold-rise change ≥ 4 per time and group.

| | 2.5 μg GLA-SE | | 5.0 μg GLA-SE | |
|---|---|---|---|---|
| **Total IgG** | **N*** | **Proportion Estimate (95% Confidence Interval)** | **N** | **ProportionEstimate (95% ConfidenceInterval)** |
| Day 0 | 0 | 0.031 (0.000, 0.118) | 0 | 0.031 (0.000, 0.118) |
| Day 28 | 1 | 0.094 (0.0002, 0.233) | 1 | 0.094 (0.0002, 0.233) |
| Day 56 | 5 | 0.344 (0.129, 0.569) | 2 | 0.156 (0.013, 0.328) |
| Day 84 | 11 | 0.719 (0.505, 0.917) | 8 | 0.531 (0.297, 0.764) |
| Day 110 | 11 | 0.719 (0.505, 0.917) | 8 | 0.531 (0.297, 0.764) |
| Day 140 | 10 | 0.656 (0.431, 0.871) | 7 | 0.469 (0.236, 0.703) |
| **IgG1** | | | | |
| Day 0 | 0 | 0.031 (0.000, 0.118) | 0 | 0.031 (0.000, 0.118) |
| Day 28 | 1 | 0.094 (0.0002, 0.233) | 0 | 0.031 (0.000, 0.118) |
| Day 56 | 6 | 0.406 (0.180, 0.639) | 2 | 0.156 (0.013, 0.328) |
| Day 84 | 12 | 0.781 (0.585, 0.957) | 8 | 0.531 (0.297, 0.764) |
| Day 110 | 11 | 0.719 (0.505, 0.917) | 6 | 0.406 (0.180, 0.639) |
| Day 140 | 10 | 0.656 (0.431, 0.871) | 3 | 0.219 (0.043, 0.415) |
| **IgG2** | | | | |
| Day 0 | 0 | 0.031 (0.000, 0.118) | 0 | 0.031 (0.000, 0.118) |
| Day 28 | 0 | 0.031 (0.000, 0.118) | 0 | 0.031 (0.000, 0.118) |
| Day 56 | 1 | 0.094 (0.0002, 0.233) | 1 | 0.094 (0.0002, 0.233) |
| Day 84 | 1 | 0.094 (0.0002, 0.233) | 3 | 0.219 (0.043, 0.415) |
| Day 110 | 0 | 0.031 (0.000, 0.118) | 1 | 0.094 (0.0002, 0.233) |
| Day 140 | 0 | 0.031 (0.000, 0.118) | 1 | 0.094 (0.0002, 0.233) |
| **IgG3** | | | | |
| Day 0 | 0 | 0.031 (0.000, 0.118) | 0 | 0.031 (0.000, 0.118) |
| Day 28 | 0 | 0.031 (0.000, 0.118) | 1 | 0.094 (0.0002, 0.233) |
| Day 56 | 4 | 0.281 (0.083, 0.495) | 2 | 0.156 (0.013, 0.328) |

| | | | | |
|---|---|---|---|---|
| Day 84 | 5 | 0.344 (0.129, 0.569) | 7 | 0.469 (0.236, 0.703) |
| Day 110 | 1 | 0.094 (0.0002, 0.233) | 3 | 0.219 (0.043, 0.415) |
| Day 140 | 0 | 0.031 (0.000, 0.118) | 2 | 0.156 (0.013, 0.328) |

| **IgG4** | | | | |
|---|---|---|---|---|
| Day 0 | 0 | 0.031 (0.000, 0.118) | 0 | 0.031 (0.000, 0.118) |
| Day 28 | 0 | 0.031 (0.000, 0.118) | 0 | 0.031 (0.000, 0.118) |
| Day 56 | 2 | 0.156 (0.013, 0.328) | 0 | 0.031 (0.000, 0.118) |
| Day 84 | 1 | 0.094 (0.0002, 0.233) | 1 | 0.094 (0.0002, 0.233) |
| Day 110 | 2 | 0.156 (0.013, 0.328) | 0 | 0.031 (0.000, 0.118) |
| Day 140 | 0 | 0.031 (0.000, 0.118) | 0 | 0.031 (0.000, 0.118) |

| **IgGE** | | | | |
|---|---|---|---|---|
| Day 0 | 0 | 0.031 (0.000, 0.118) | 0 | 0.031 (0.000, 0.118) |
| Day 28 | 1 | 0.094 (0.0002, 0.233) | 0 | 0.031 (0.000, 0.118) |
| Day 56 | 0 | 0.031 (0.000, 0.118) | 0 | 0.031 (0.000, 0.118) |
| Day 84 | 3 | 0.219 (0.043, 0.415) | 0 | 0.031 (0.000, 0.118) |
| Day 110 | 0 | 0.031 (0.000, 0.118) | 0 | 0.031 (0.000, 0.118) |
| Day 140 | 1 | 0.094 (0.0002, 0.233) | 0 | 0.031 (0.000, 0.118) |

* N = number of individuals with fold-rise change ≥ 4
