# Supplementary figures and images for: The Sm14+GLA-SE Recombinant Vaccine Against Schistosoma mansoni and S. haematobium in Adults and School Children: Phase II Clinical Trials in West Africa
Source: Vaccines (Basel). 2025 Mar 16;13(3):316. doi: 10.3390/vaccines13030316 (PMC11946331; doi:10.3390/vaccines13030316)

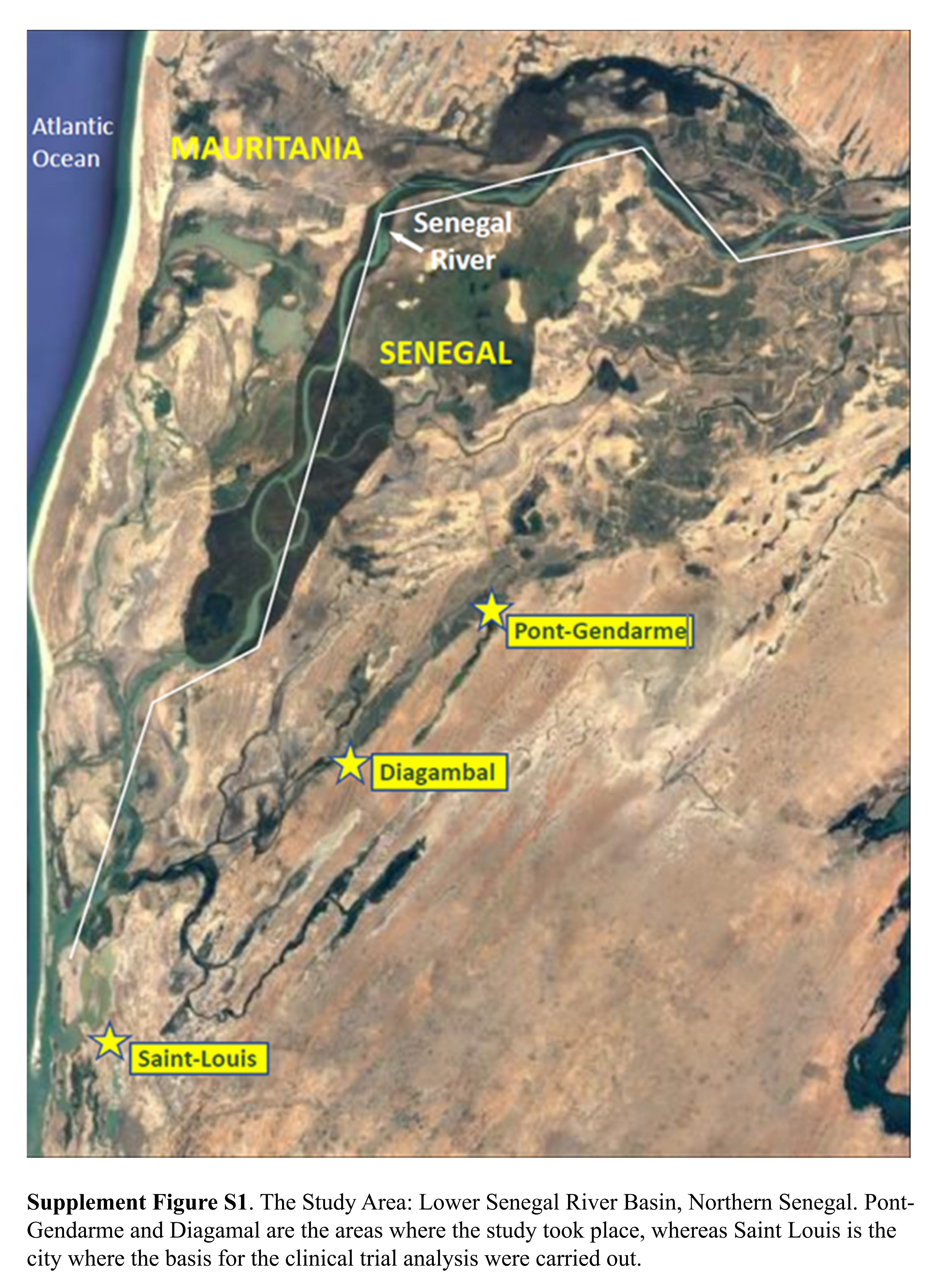

Supplement: Supplementary file 1 [file vaccines-13-00316-s001.zip › Figure S1.jpg]

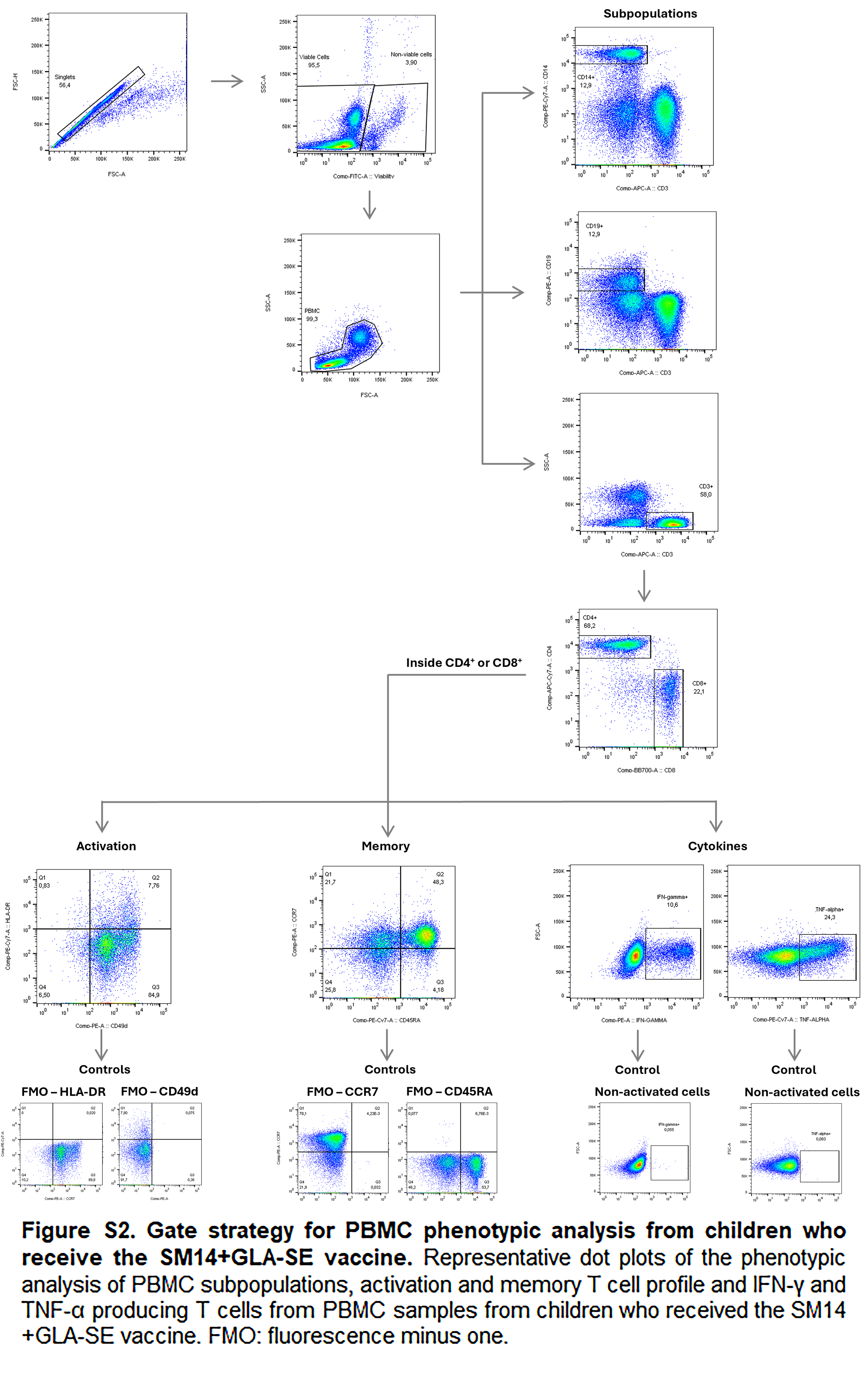

Supplement: Supplementary file 1 [file vaccines-13-00316-s001.zip › Figure S2.png]
